# Pregnane X-receptor promotes stem cell-mediated colon cancer relapse

**DOI:** 10.18632/oncotarget.10646

**Published:** 2016-07-18

**Authors:** Chris Planque, Fatemeh Rajabi, Fanny Grillet, Pascal Finetti, François Bertucci, Meritxell Gironella, Juan José Lozano, Bertrand Beucher, Julie Giraud, Véronique Garambois, Charles Vincent, Daniel Brown, Ludovic Caillo, Jovana Kantar, André Pelegrin, Michel Prudhomme, Jérémie Ripoche, Jean François Bourgaux, Christophe Ginestier, Antoni Castells, Frédéric Hollande, Julie Pannequin, Jean Marc Pascussi

**Affiliations:** ^1^ CNRS UMR5203, Institut de Génomique Fonctionnelle, Montpellier, France; ^2^ INSERM U1191, Montpellier, France; ^3^ Université Montpellier, Montpellier, France; ^4^ Centre de Recherche en Cancérologie de Marseille, INSERM UMR1068, CNRS UMR725, Marseille, France; ^5^ Centro de Investigación Biomédica en Red de Enfermedades Hepáticas y Digestivas (CIBEREHD), Institut d'Investigaciones Biomèdiques August Pi i Sunyer (IDIBAPS), Barcelona, Spain; ^6^ Institut de Recherche en Cancérologie de Montpellier, Montpellier, France; ^7^ Department of Pathology, University of Melbourne, Parkville, Australia; ^8^ Laboratoire de Biochimie, CHU Carémeau, Nîmes, France; ^9^ Service de Chirurgie Digestive, CHU Carémeau, Nîmes, France; ^10^ Service d'Hépato-Gastroentérologie, CHU Carémeau, Nîmes, France; ^11^ Centre de Recherche en Cancérologie de Marseille, U1068 Inserm, Marseille, France

**Keywords:** PXR, colorectal cancer, cancer stem cell, tumor recurrence

## Abstract

Colorectal cancer lethality usually results from post-treatment relapse in the majority of stage II-IV patients, due to the enhanced resistance of Cancer Stem Cells (CSCs). Here, we show that the nuclear receptor Pregnane X Receptor (PXR, NR1I2), behaves as a key driver of CSC-mediated tumor recurrence. First, PXR is specifically expressed in CSCs, where it drives the expression of genes involved in self-renewal and chemoresistance. Clinically, high levels of PXR correlate with poor recurrence-free survival in a cohort of >200 stage II/III colorectal cancer patients treated with chemotherapy, for whom finding biomarkers of treatment outcome is an urgent clinical need. shRNA silencing of PXR increased the chemo-sensitivity of human colon CSCs, reduced their self-renewal and tumor-initiating potential, and drastically delayed tumor recurrence in mice following chemotherapy. This study uncovers PXR as a key factor for CSC self-renewal and chemoresistance and targeting PXR thus represents a promising strategy to minimize colorectal cancer relapse by selectively sensitizing CSCs to chemotherapy.

## INTRODUCTION

Colorectal cancer (CRC) is the third most commonly diagnosed cancer, which causes 655,000 deaths annually worldwide [1]. To this day systemic chemotherapy cocktails (5-fluorouracil (5-FU), irinotecan, and oxaliplatin) that target the bulk population of proliferative tumor cells remain the cornerstone of treatment for advanced CRC [2]. However, treatment efficiency is severely hampered by the frequent occurrence of drug-resistance and post-treatment tumor recurrence. In recent years, highly tumorigenic sub-populations of cancer cells, named Cancer Stem Cells (CSCs), have been implicated in post-treatment tumor recurrence [3]. These cells are characterized by the expression of stem cell factors that endow them with stemness properties, such as the ability to self-renew. They are capable of initiating and sustaining tumor growth in serial transplantation assays [4, 5]. In addition, they are notably characterized by a higher capacity to resist treatments compared to other tumor cells [6, 7]. Evidence from xenograft models [8] and human trials [9] indicate selective enrichment of CSCs in CRC tumors that survive therapy.

Given that CSCs are the chief culprits in the failure of current therapies, it is important to identify innovative approaches that target them to improve clinical outcomes for cancer patients. To date, most strategies aim to inhibit CSC self-renewal or to induce their differentiation [10]. However, these phenotypic traits are shared by healthy adult stem cells, leading to specific concerns about potential side effects. An alternative CRC therapy would be to sensitize CSCs to current therapies, but the precise mechanism underlying the higher drug resistance of CSCs remains unclear. Multiple parameters have been proposed to be involved in CSC drug resistance, including slow proliferation, increased resistance to DNA damage, activation of anti-apoptosis mechanisms, but also expression of multidrug transporters such as ATP-binding cassette G2 (ABCG2 [11]) and of drug metabolizing enzymes including aldehyde dehydrogenase 1A1 (ALDH1A1 [12]) and the cytochrome P450 CYP3A4 [13]. Accordingly, flow cytometry strategies based on ABCG2 expression (Side Population, SP) and ALDH-activity (Aldefluor-‘bright’ cells or ALDH^br^) have been used to identify CSCs in various types of solid tumors including CRC [14, 15]. SP cells and ALDH^br^ cells are also characterized by an enhanced chemoresistance to various cytotoxics [16, 17, 8]. Consequently, these populations are enriched following chemotherapy [8, 16].

We previously reported that the orphan nuclear receptor PXR (Pregnane X Receptor, NR1I2), a key regulator of xenobiotic metabolism in the liver [18], increased irinotecan resistance in CRC cell lines [19]. Since PXR has been previously described to regulate CYP3A4 gene expression in human hepatocytes [18], and Aldh1a1 and Abcg2 genes in mice [20] and porcine [21] tissues respectively, we hypothesized that PXR may participate in the intrinsic chemoresistance of CSCs. Here, we demonstrate that PXR expression and activity are indeed highly restricted to CSCs, where it drives the expression of a large network of genes that are instrumental for CSC chemoresistance *in vitro* and *in vivo*. Furthermore, we observed that PXR and its targets form a prognostic indicator for CRC relapse. These results pinpoint PXR as a clinically druggable Achilles' heel for CSCs in colorectal and potentially also in other cancers.

## RESULTS

### PXR expression and activity define chemoresistant colon cancer stem cells

**Figure 1:**
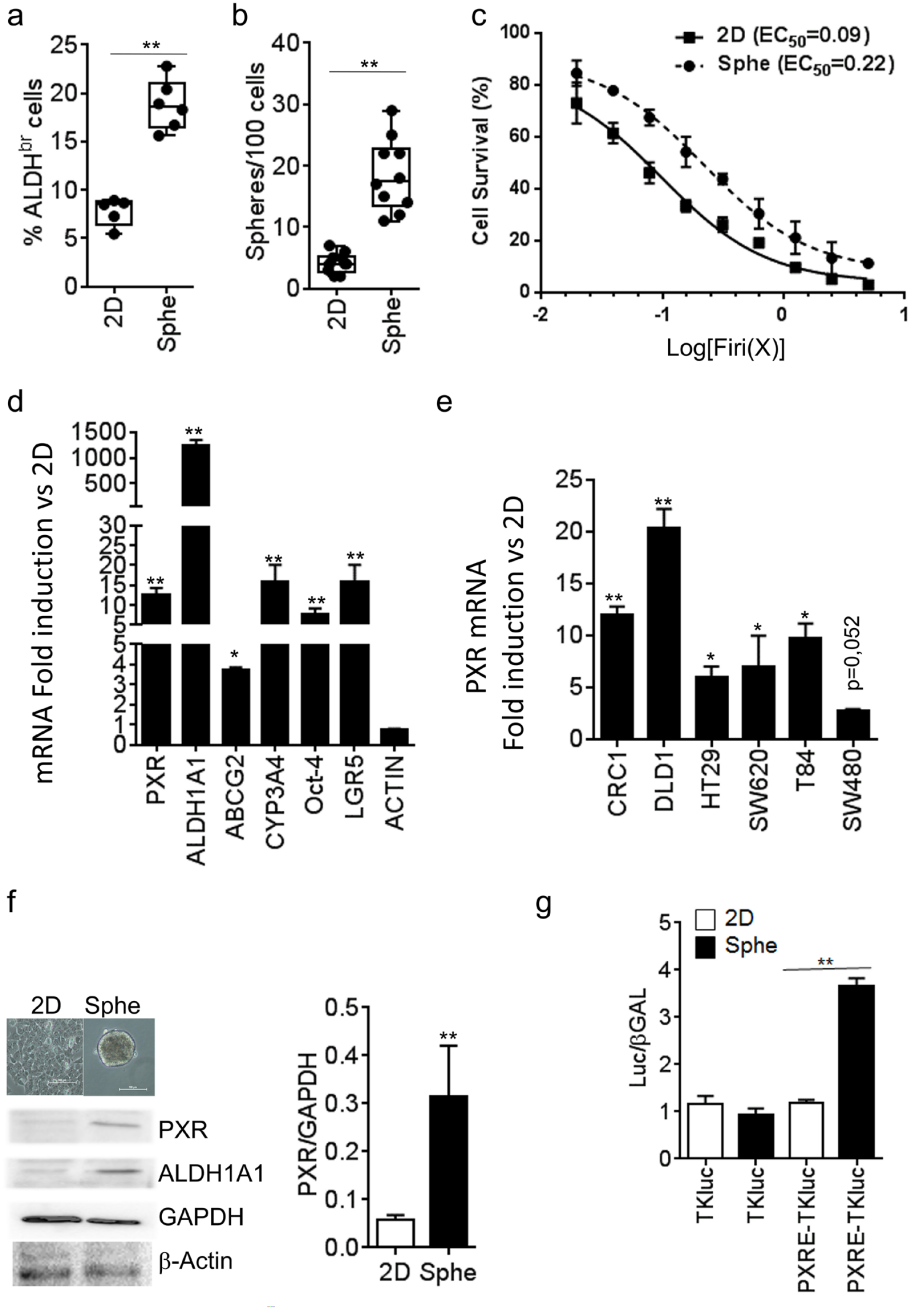
PXR expression is increased in colorectal spheroid models **a.** Percentage of ALDH^br^ cells, and **b.** percentage of Sphere-Forming Cells of patient-derived CRC cells (CRC1) grown as adherent (2D) or colonosphere (Sphe) cultures. **c.** Percentage of CRC1 surviving cells 72 hours after exposure to the indicated concentrations of Firi (1X=50μM 5-FU + 500nM SN38). Data are expressed as mean ± SEM of 3 experiments, and half maximal effective concentrations (EC_50_) are indicated. **d.** RT-qPCR analyses of mRNA expression for PXR, PXR target and CSC marker genes in CRC1 cells maintained as Spheroids compared to 2D conditions (F.I., Fold Induction). **e.** RT-qPCR quantification of PXR mRNA in CRC cell lines maintained as Spheroids compared to 2D conditions. *d,e,:* Data are expressed as mean ± SEM (n>3) and reported as fold change compared to cells grown in 2D conditions. **f.** Western-blot analysis of PXR expression in CRC1 cells maintained as Spheroids compared to 2D conditions. Data are expressed as mean ± SEM (n=3) of PXR/GAPDH ratio. **g.** PXR transcriptional activity was determined in CRC1 cells after transfection with luciferase reporter plasmids. Data are expressed as mean ± SEM (n=3) of luciferase/β-Galactosidase ratio, normalized to 2D conditions. *, p<0.05; **, p<0.005.

We compared PXR expression in CSCs *versus* non-CSCs by using enrichment of self-renewing cells *(i.e*. spheroids passaging). Previous studies showed that under defined conditions, CRC cells maintained as floating spheroids display enhanced expression of cancer stem cell markers, increased self-renewal and resistance to chemotherapeutic drugs [16]. We confirmed those observations in patient-derived CRC cells (CRC1) maintained as spheroids (Sphe) compared to cells maintained in adherent conditions and exposed to serum-containing medium (2D). As shown, cells isolated from spheroids had a higher proportion of cells with ALDH activity (Aldefluor positive (‘bright’) cells, ALDH^br^, Figure 1a). In addition, they had enhanced spheroid-forming efficiency (Figure 1b), a widely used *in vitro* assay for CSC quantification [7], calculated as the percentage of seeded cells that gave rise to a spheroid. Moreover, cells maintained as spheroids had a greater resistance to a combination of 5-FU and SN38 (hereafter named “Firi” as SN38 is the active metabolite of irinotecan), when compared to the same cells maintained as adherent monolayers (≈2-fold increase in EC_50_, p<0.01; Figure 1c). RT-qPCR analyses revealed the higher expression of PXR in spheroids compared to 2D conditions as well as an enriched expression of colon CSCs markers (ALDH1A1, Oct-4 [22] and LGR5 [23]) and PXR target genes (CYP3A4 and ABCG2) (Figure 1d). Similar results were obtained in other tested human CRC cell lines (Figure 1e) and patient-derived CRC cells (Supplementary Figure S1a). In addition, both PXR protein expression and PXR transcriptional activity, measured using a PXR luciferase reporter gene [24], were higher in spheroids compared to adherent (2D) conditions (Figure 1f, 1g). Finally, serial passaging of spheroids (passages 1 to 8, i.e. S1 to S8) resulted in the gradual and concomitant enrichment of spheroid-forming efficiency, of ALDH^br^ cell percentage and of mRNA expression of CSC markers, correlating tightly with the up regulation of PXR (Supplementary Figure S1b-S1d). Taken together these findings indicate that PXR is preferentially expressed and active in self-renewing colon CSCs.

**Figure 2:**
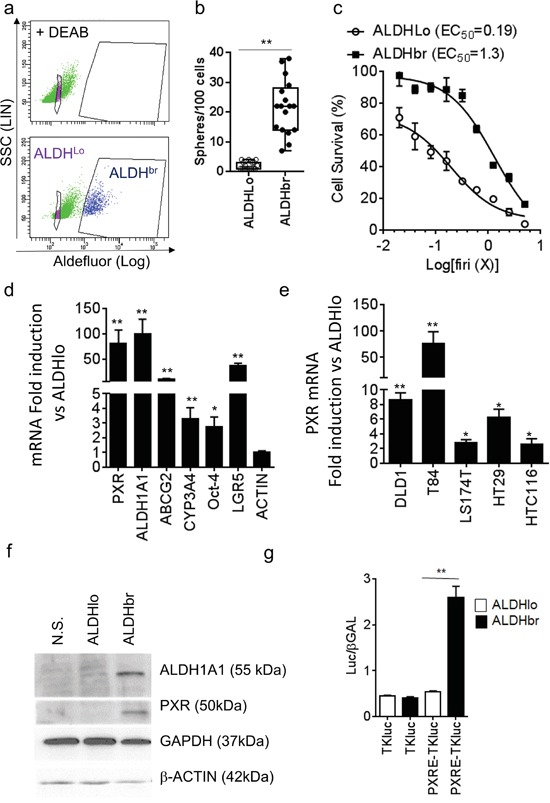
PXR expression is increased in colorectal ALDH^br^ CSCs **a.** Aldefluor assay and gates used for CRC1 cell-sorting. The ALDH inhibitor DEAB was added to identify ALDH-positive (ALDH^br^) cells. **b.** Percentage of sphere-forming cells of the ALDH^br^ and ALDH^lo^ populations. **c.** Percentage of surviving cells, 72 hours after exposure of ALDH^br^ and ALDH^lo^ cells to the indicated concentrations of Firi (1X=50μM 5-FU + 500nM SN38). Data are expressed as mean ± SEM of 3 experiments, and half maximal effective concentrations (EC_50_) are indicated. **d.** RT-qPCR analyses of mRNA expression for PXR, PXR targets and CSC marker genes in the ALDH^br^ compared to the ALDH^lo^ population (F.I., Fold Induction). **e.** RT-qPCR quantification of PXR mRNA in ALDH^br^ and ALDH^lo^ cells from CRC cell lines. *d,e:* Data are expressed as mean ± SEM (n=3) and reported as fold change compared to ALDH^lo^ cells. **f.** ALDH1A1, PXR, GAPDH and β-ACTIN protein expression in unsorted (NS), ALDH^lo^ and ALDH^br^ CRC1 cells. **g.** PXR transcriptional activity was determined in ALDH^lo^ and ALDH^br^ CRC1 cells 24 hours after transfection with luciferase reporter plasmids. Data are expressed as mean ± SEM (n=3) of luciferase/β-Galactosidase ratio. *, p<0.05; **, p<0.005.

We then isolated CSCs using fluorescence-activated cell sorting based on the activity of Aldefluor. Aldefluor activity and ALDH1A1 gene expression have been described as colon CSC markers [12, 25] and the latter is a regulator of chemoresistance [17, 26, 27] and cell renewal [28]. First, we confirmed that the enhanced Aldefluor activity (ALDH^br^, Figure 2a) was indeed associated with the specific expression of the ALDH1A1 mRNA isoform in CRC cells (Supplementary Figure S2a, S2b). CRC1 ALDH^br^ cells had greater spheroid-forming efficiency (Figure 2b) and they were more resistant to Firi treatment (≈6.8-fold higher EC_50_, p<0.01; Figure 2c) than ALDH^lo^ cells. As shown on Figure 2d, PXR mRNA was strongly enriched in the ALDH^br^ population. Similar results were obtained in other tested CRC cell lines (Figures 2e and Supplementary Figure S1b). PXR protein expression was only detected in the ALDH^br^ population (Figure 2f), which expectedly had higher ALDH1A1 mRNA and protein levels (Figures 2d, 2f, and Supplementary Figure S2a, S2b). In addition, luciferase reporter genes for PXR transcriptional activity were specifically increased in ALDH^br^ cells (Figures 2g and Supplementary Figure S2c, S2d), indicating that PXR is preferentially expressed and active in colon ALDH^br^ cells.

**Figure 3:**
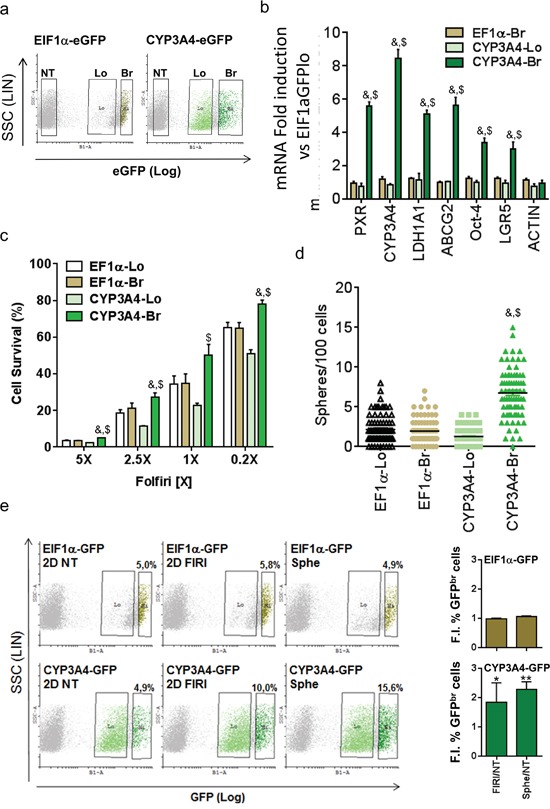
PXR transcriptional activity marks chemoresistant CSCs **a.** Flow cytometry profiles of EIF1α-eGFP and CYP3A4-eGFP infected T84 cells and regions used for cell-sorting. NT= Non infected, Lo=GFP low, Br=GFP bright. **b.** RT-qPCR analyses of PXR, PXR target and CSC marker gene mRNA expression in *Br* and *Lo* populations compared to EIF1α-GFP low cells (F.I., Fold Induction). Data are expressed as mean ± SEM (n=3). **c.** Percentage of surviving cells, 72 hours after exposure of sorted cells to the indicated dilutions of Firi (1X=50μM 5-FU + 500nM SN38). Data are expressed as mean ± SEM (n=4). **d.** Percentage of sphere-forming cells for *Br* and *Lo* populations. a,b,c: *#*: p<0.05 compared to *Lo* cells for each GFP construct, *$*: p<0.05 compared to EIF1α-GFP Br cells. **e.** Representative flow cytometry profiles of EIF1α-eGFP- and CYP3A4-eGFP infected cells before (2D NT) or after 72h of Firi (50μM 5-FU + 500nM SN38) treatment followed by 2 days of recovery without treatment (2D FIRI) or maintained as colonospheres (Sphe). The fold increase in GFP *Br* percentage induced by Firi (2D FIRI/2D NT) or colonosphere conditions (Sphe/2D NT) are indicated for each GFP construct as mean ± SEM (n>3). *, p<0.05; **, p<0.005.

To validate the preferential expression and activity of PXR in chemoresistant CSCs, we infected T84 cells with a GFP-tagged PXR-driven promoter (CYP3A4eGFP) to selectively sort cells according to their PXR activity level (Figure 3a); cells transduced with an EIF1α -driven eGFP construct were used as control. We first validated the reporter system by comparing the expression of PXR and its target genes in cell populations sorted according to their GFP expression level. Cells with high PXR activity (i.e. CYP3A4eGFP^br^) had higher PXR, CYP3A4 and ABCG2 mRNA expression (Figure 3b). Furthermore, CYP3A4eGFP^br^ cells had enhanced expression of colon CSC markers such as ALDH1A1, Oct-4 and LGR5. In addition, CYP3A4eGFP^br^ cells had higher resistance towards Firi treatment (Figure 3c). Finally, CYP3A4eGFP^br^ cells had higher sphere-forming efficiency (Figure 3d) and a ten-fold higher stem cell frequency (as calculated according to Hu et al. [29]) (1/9±3 for CYP3A4^br^ cells vs 1/94±32 for CYP3A4^lo^ cells, p=0.002) compared to CYP3A4eGFP^lo^ cells. Accordingly, flow cytometry analyses showed a specific increase of cells with strong PXR activity after CSC selection, by spheroid passaging or Firi treatment (Figure 3e). Together these data demonstrate that PXR transcriptional activity tracks chemo-resistant CSCs.

### PXR regulates a large network of CSC resistance genes and of poor prognosis factors in colon cancer patients treated with chemotherapy

**Figure 4:**
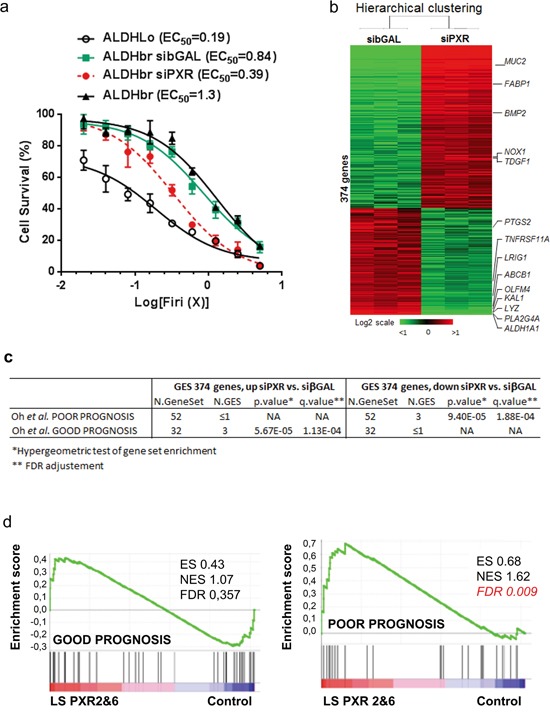
PXR regulates a large network of key CSC chemoresistance genes **a.** Percentage of surviving cells, 48 hours after exposure of cells to the indicated concentrations of Firi (1X=50μM 5-FU + 500nM SN38). ALDH^br^ CRC1 cells were first transfected with or without 100nM of control (siβGAL) or PXR-specific (siPXR-1334) siRNA. Data are expressed as mean ± SEM (n=3). **b.** Hierarchical clustering of 6 samples and 374 genes, differentially expressed between PXR siRNA (siPXR) and control siβGAL-transfected ALDH^br^ CRC1 cells. Each row represents a gene and each column represents a sample. **c,d.** Gene set enrichment analyses (GSEA) were used to interrogate the similarity of genes that were differentially expressed in the microarray experiments after loss- (c) or gain-of PXR (d) function, to signatures of patient prognosis (Oh, S.C., et al. Gut 61, 1291-1298 (2012)). *c:* genes induced by siRNA-mediated PXR depletion in ALDHbr cells “Up siPXR vs. siβGAL” or decreased by PXR depletion “Down siPXR vs. siβGAL”. *d:* PXR-overexpressing LS174T clones 2&6 versus LS174T control.

Having established the preferential expression and activity of PXR in colon CSCs, we then directly assessed the impact of PXR down-regulation on both chemoresistance and mRNA expression profile of patient-derived ALDH^br^ cells transfected with PXR siRNA (Supplementary Table S1 for siRNA sequences). First, we observed that ALDH^br^ cell survival was significantly decreased after transfection with an efficient siRNA sequence decreasing PXR expression (see Supplementary Figure S5a for validation): ALDH^br^ siPXR EC_50_=0.40 compared to ALDH^br^ siβGAL EC_50_=0.83 (p<0.01, Figure 4a). Microarray experiments showed that, 48 hours after transfection, the expression of 374 genes was significantly altered by PXR down-regulation in ALDH^br^ cells (Supplementary Table S2 and Figure 4b). Notably, PXR-depletion led to the down-regulation of CSC markers such as ALDH1A1 [12], OLFM4 [30], and LRIG-1 [31] and the up-regulation of differentiation markers such as MUC2 (goblet cells) and FABP1 (enterocytes). A Gene Ontology analysis highlighted that PXR depletion in ALDH^br^ cells down-regulates the DNA damage sensor and repair machinery (known to help CSCs to overcome many standard anticancer treatments [32]), while it promotes the expression of genes involved in apoptosis and cell death (Supplementary Figure S3). GSEA analyses based on a clinical study describing outcome-related gene signatures in groups of patients homogeneously treated with 5-FU-based chemotherapy [33], showed that down-regulated genes in ALDH^br^ cells, following PXR depletion, were specifically enriched in a poor progression-free survival signature. Conversely, genes that were up-regulated in PXR-depleted cells were enriched in the good-prognosis signature (Figure 4c). Similar results were observed after PXR gain-of function in two different LS174T clones (i.e. LS PXR2 and LS PXR6; Supplementary Figure S4a) in which microarray (Supplementary Table S3) and RT-qPCR validation analyses (Supplementary Figure S4b) confirmed that over-expression of PXR regulates the expression of multiple colon CSC markers (ALDH1A1, ABCG2, CYP3A4, CD24 [34], CXCR4 [35], LRIG-1 and OLFM4) and prognostic markers for colorectal tumor recurrence (ABCC6 [36] and S100A10 [37]). Again, GSEA analyses showed that genes with significantly higher expression levels in LS PXR2 and LS PXR6 cells were specifically enriched in the poor-prognosis signature [33] (Figure 4d). Together these results indicate that PXR controls the expression of a large gene network associated with CSC phenotype and post-chemotherapy tumour recurrence.

**Figure 5:**
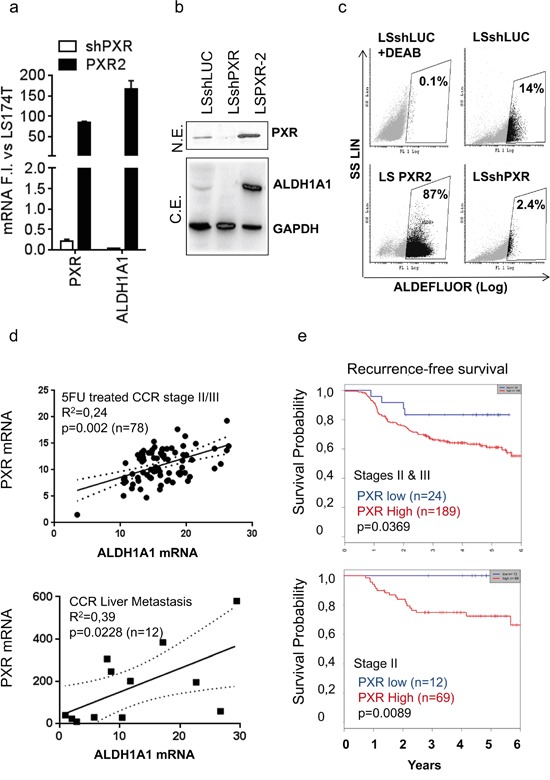
PXR mRNA expression correlates with ALDH1A1 expression and is a poor prognosis factor in colon cancer patients **a.** RT-qPCR analyses of PXR and ALDH1A1 mRNA expression, **b.** detection of PXR, ALDH1A1 and GAPDH protein expression by Western blotting after loss- (shPXR) or gain of PXR expression (PXR2). *a:* Data are expressed as mean ± SEM (n≥3) compared to LS174T control cells (shLUC) (F.I., Fold Induction). *b:* N.E.= nuclear extracts, C.E.= cytosolic extracts. **c.** Quantification of ALDH activity using the Aldefluor assay. Percentages of ALDH^br^ cells are indicated in inset boxes. **d.** Correlation between PXR and ALDH1A1 mRNA expression in CRC primary tumors of stage II or III patients treated with 5-FU-based chemotherapy (n=78) or in human liver metastasis samples (n=12) obtained from patients with metastatic colorectal disease. **e.** Kaplan-Meier estimates of the probability of resting free from tumor recurrence, according to PXR expression level in CRC primary tumors of stage II & III (upper panel), or stage II (lower panel) patients treated with 5-FU-based chemotherapy. PXR expression was normalized according to RPLO mRNA expression. Patients with low PXR gene expression are depicted in blue and patients with high PXR gene expression in red. *p* value from the likelihood ratio test is shown in each case.

Since ALDH1A1 appeared as one of the top-ranked differentially regulated genes after PXR up-regulation or inhibition (Supplementary Tables S2&S3) and has been described as key CSC marker [12, 25] and a poor prognosis factor in colon cancer patients [38], we focused our attention on its putative regulation by PXR. First, gain- and loss-of function in LS174T cells confirmed that the expression of ALDH1A1 mRNA and protein was positively regulated by PXR (Figure 5a, 5b). In addition, the proportion of ALDH^br^ cells was enhanced in PXR-overexpressing cells and strongly decreased upon PXR down regulation by shRNA (Figure 5c). Similar results were obtained in the T84 CRC cell line (Supplementary Figure S4c, S4d, S4e). Experiments using different shRNA sequences leading to the specific down-regulation of PXR expression or rescue experiment confirmed the direct involvement of PXR on ALDH1A1 and Aldefluor activity level (Supplementary Figure S5). To assess the clinical relevance of correlation between PXR and ALDH expression, we compared PXR and ALDH1A1 mRNA expression in human samples. As shown, PXR and ALDH1A1 were significantly correlated in primary tumor samples from stage II & III CCR patients (n=78) and in CRC liver metastatic samples (n=12) (Figure 5d). In addition, we attempted to determine whether PXR expression was correlated with clinical outcome in homogenous groups of chemotherapy-treated colorectal cancer patients. We first performed a Kaplan-Meier analysis of PXR mRNA expression in 5-FU-treated stage II/III CRC patient cohort [37]. We found that patients bearing tumors with high PXR expression had a significantly lower probability of disease-free survival, indicating that high PXR is associated with poor prognosis in stage II/III colon cancer patients treated with 5-FU-based chemotherapy (n=213; p=0.0369; Figure 5e). This effect was even more markedly increased when we analysed the outcome for stage II patients only (n=81; p=0.0089), for whom finding biomarkers of treatment outcome is an urgent clinical need. Similar results were obtained by using RPLO (Figure 5e) or GAPDH (Supplementary Figure S6) as reference gene. These results clearly demonstrated that elevated PXR expression in CCR patients is positively correlated with a higher recurrence frequency, an outcome that is often driven by the persistence of chemoresistant cancer stem cells.

### PXR knockdown increases recurrence-free survival in mouse xenograft models

**Figure 6:**
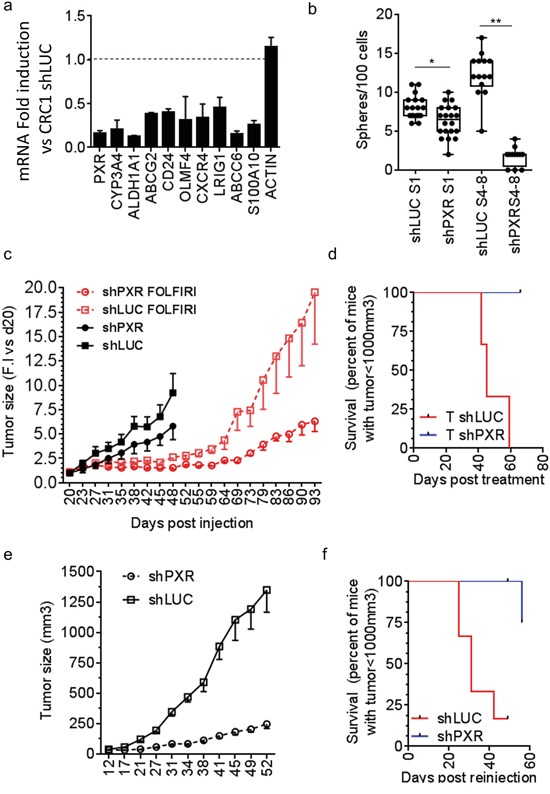
Knock-down of PXR decreases CSC-self renewal and improves recurrence free survival in mouse xenograft models **a.** RT-qPCR analyses of PXR and PXR target gene mRNA expression in CRC1 cells expressing PXR-shRNA (shPXR-1334). Data are expressed as mean ± SEM (n=3) compared to control CRC1 cells (CRC1 shLUC). *, p<0.05; **, p<0.005. **b.** Percentage of sphere-forming cells of shLuc and shPXR CRC1 colon cancer cells maintained as first-generation spheres (S1) or after 4-8 passages (S4-S8). Data are expressed as mean ± SEM (n=3). **c.** Tumor volume over time after subcutaneous injection of 15,000 shLuc or shPXR CRC1 colon cancer cells isolated from colonospheres (18mice/group). Mice were randomized once tumors reached a volume of 100 mm^3^ (day 20). Three days after randomization, one group (6 mice/group) was treated twice weekly for 4 weeks with vehicle or with Folfiri. At day 48, three days after the end of treatment, 6 mice per group were sacrificed and the remaining ones from the Folfiri-treated groups (6 mice/group) were kept for 7 weeks without treatment to further monitor tumor growth. Data are expressed as mean ± SEM of the fold-increase (F.I.) compared to the corresponding tumor volume at day 20. **d.** Kaplan–Meier survival plots after treatment cessation representing the time for animals to reach the endpoint tumor size of 1000 mm3. **e.** Tumor volume over time after subcutaneous injection of tumor cells obtained at day 48 from xenograft tumors shown in *(c)*, processed for cell dissociation and sub-cutaneously re-implantated in immunodeficient mice (6 mice/group, 1,500 live (7-AAD-negative) cells/mouse). **f.** Survival plots after secondary injection of residual Folfiri-treated shLUC or shPXR cells.

To study the relationship between PXR and CSCs chemoresistance, we established spheroid cultures from patient-derived colorectal tumor samples (CRC1) and infected these cells with a lentivirus allowing the stable expression of small hairpin RNAs targeted against PXR (shPXR) or a control (shLUC). A significant down-regulation of PXR and PXR-regulated gene mRNAs was observed in shPXR-expressing spheroids (Figure 6a). PXR depletion reduced both the ALDH^br^ cell population (Supplementary Figure S7) and sphere-forming potential (Figure 6b) and these effects were strongly amplified following serial spheroid passaging. To determine the impact of PXR on CSC chemoresistance and post-treatment relapse, we generated tumor xenografts by subcutaneously injecting 15,000 PXR-deficient (shPXR) or control (shLUC) cells from spheroids into nude mice (n=18/group). Twenty days after injection, mice were randomized and received either vehicle or a Folfiri regimen (90mg/kg leucovorin, 50mg/kg 5-FU and 30mg/kg irinotecan, twice a week from day 23 to 45) designed to induce tumor regression [39]. Tumor growth was noticeably slower in shPXR group compared to shLUC control group in absence of treatment and tumor stasis upon Folfiri treatment was detected in both groups (Figure 6c). Three days after treatment cessation (day 48), six mice from the vehicle- and Folfiri-treated groups were sacrificed for subsequent analysis, while tumor growth and post-treatment relapse were monitored in the remaining mice. Recurrence was clearly noticeable from 3 weeks post-treatment (day 69) in shLUC control group, which had a ≈2-fold accelerated growth rate compared to untreated tumors. In contrast, the growth rate of shPXR tumors did not increase after Folfiri treatment (Figure 6c) and their recurrence was strongly delayed (Log-Rank Mantel-Cox test p=0.029; Figure 6d). Thus PXR depletion efficiently prolongs progression-free survival following chemotherapy. Finally, to determine whether PXR down-regulation affected the *in vivo* tumor-initiating potential of Folfiri-resistant tumor cells, we subcutaneously transplanted 1,500 cells obtained from residual tumors collected three days after treatment completion (day 48) into new nude mice (Figure 6e). Cells isolated from Folfiri-treated shLuc control tumors induced the rapid formation of second-generation tumors in all grafted animals. In contrast, tumor growth rate was much lower in mice injected with cells isolated from Folfiri-treated shPXR tumors and tumor incidence was strongly delayed (Log Rank Mantel-Cox test p=0.026; Figure 6f). These results clearly indicate that shRNA silencing of PXR delays tumor relapse and affects *in vivo* tumor–initiating potential of CRC cells following chemotherapy.

### PXR depletion impairs chemotherapy-induced enrichment of tumorigenic CSCs *in vivo*

**Figure 7:**
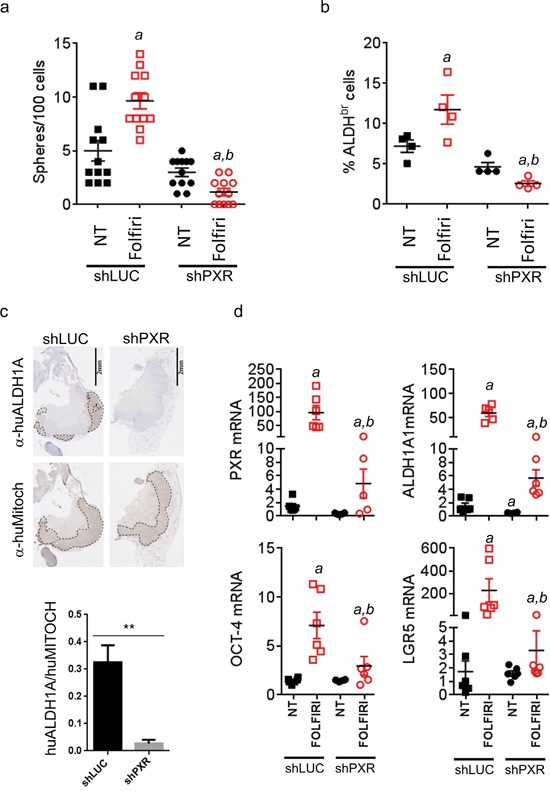
PXR depletion impairs chemotherapy-induced enrichment of PXR and CSC markers *in vivo* Tumor samples from mice sacrificed at day 48 (see Figure 6c) were processed for cell dissociation and, live (7-AAD-negative) tumor cells were further analyzed for their **a.** ability to initiate sphere formation *in vitro* and **b.** Aldefluor activity (% of ALDH^br^ cells). Data are expressed as mean±SEM. “*a*”, p<0.05 compared to non-treated shLUC tumors and “*b*”, p<0.05 compared to Folfiri-treated shLuc tumors. **c.** Hematoxylin/Eosin and immunostaining of Folfiri-treated shLuc and shPXR paraffin-embedded tumor sections (day 48) using antibodies directed against human ALDH1A (α-huALDH1A, detecting ALDH1A1 protein [54–56]) or human mitochondria (α-huMitoch). Ratios of ALDH1A/human mitochondria-stained areas on tumor sections (n=4/group) are presented below. Data are expressed as mean±SEM. **, p<0.005 **d.** Expression of the indicated mRNAs was quantified by RT-qPCR on cells isolated from dissociated tumors (day 48) of non-treated or Folfiri-treated mice. Data are expressed as mean±SEM. “*a*”, p<0.05 compared to non-treated shLUC tumors and “*b*”, p<0.05 compared to Folfiri-treated shLuc tumors.

To understand the cellular mechanism involved in the tumor relapse of CRC cells following chemotherapy, we analyzed the reservoir of CSCs from tumors, collected three days after treatment cessation (Figure 6c, day 48). We first analyzed the frequency of CSCs from tumors collected at day 48 from untreated and folfiri-treated mice. In agreement with the literature [8], we found a significant increase of spheroid-forming efficiency after Folfiri treatment in control tumors (Figure 7a), suggesting that chemotherapy increased the proportion of CSCs whilst eliminating the more differentiated and proliferative tumor cells. However, this selection of CSCs was completely abolished in shPXR-derived tumors. Indeed, spheroid-forming efficiency was even lower in shPXR-derived tumors cells following chemotherapy compared to untreated shPXR or shLUC tumor cells, therefore the down-regulation of PXR completely prevents chemotherapy-induced CSC enrichment. Accordingly, while the proportion of ALDH^br^ cells was significantly increased in Folfiri-treated control tumors, this enrichment was completely impaired in tumors derived from Folfiri-treated shPXR group (Figure 7b). Moreover, ALDH1A1-positive cells were barely detectable by immunohistochemistry in sections of Folfiri-treated tumor xenografts in the shPXR group compared to control group (Figure 7c). In addition, RT-qPCR revealed a large increase of PXR and CSC gene marker (ALDH1A1, Oct-4 and LGR5) mRNA expression after Folfiri treatment of control tumors (Figure 7d). These increases were severely impaired in shPXR tumors. Comparable results were obtained *in vitro* in patient-derived cells (CRC1) or in the LS174T cell line (Supplementary Figure S8) treated with a combination of 5FU and SN38. These results suggest that PXR signaling plays an instrumental role in CSC resistance and that PXR inhibition minimizes the clinically problematic enrichment of these cells in response to chemotherapy.

## DISCUSSION

PXR is a nuclear receptor whose downstream target genes are involved in the production of phase I and II metabolic enzymes and phase III drug transporters. PXR activation has been implicated in poor response of cancer cells [40]. Here we show that the activity of this receptor is largely restricted to the minority self-renewing cell-subpopulation of tumors and that it is responsible for the ability of cancer stem cells to successfully withstand sustained chemotoxic aggression and this drives post-treatment tumor recurrence. First, we observed that PXR expression is higher in CSC-enriched tumor cells. PXR expression was enriched during spheroid passaging (Figures 1 and Supplementary Figure S1), after cell sorting using Aldefluor activity (Figures 2 and Supplementary Figure S2) and also after drug selection in several CCR cell lines and patient-derived cells (Figures 7, Supplementary Figure S8, S9 and S10). In addition, CRC cells with enhanced PXR transcriptional activity had increased expression of CSC markers, self-renewal and chemoresistance (Figure 3). These results are in agreement with the fact that, PXR was one of the most overexpressed nuclear receptors in human glioma stem cells compared to glioma cells [41].

By using both gain- and loss- of function approaches, we show that PXR drives the expression of a large number of genes that promote several hallmarks of cancer stem cells, such as chemoresistance, DNA repair and self-renewal (Supplementary Tables S2 and S3). Notably, we observed that PXR controls the expression of genes that were previously reported to confer CSC chemoresistance or to have a negative impact on disease-free survival in colon cancer patients, including ABCG2, ABCC6, ALDH1A1, CYP3A4, or S100A10. These observations were corroborated by our observation that stage II and III CRC patients with high PXR expression have a lower probability of disease-free survival after chemotherapy (Figures 5 and Supplementary Figure S6), while GSEA analyses showed that PXR target genes are associated with a poor-prognosis molecular signature in colorectal cancer patients (Figure 4). These data strongly suggest that PXR is an upstream molecular switch in CSCs that drives a network of key downstream targets that enable them to resist treatment. PXR may promote the adaptation of CSCs to their environment by providing them with a multi-faceted arsenal that includes an enhanced capacity to resist cell death and chemotoxic insult and thus the ability to self-renew and maintain their stemness potential.

**Figure 8:**
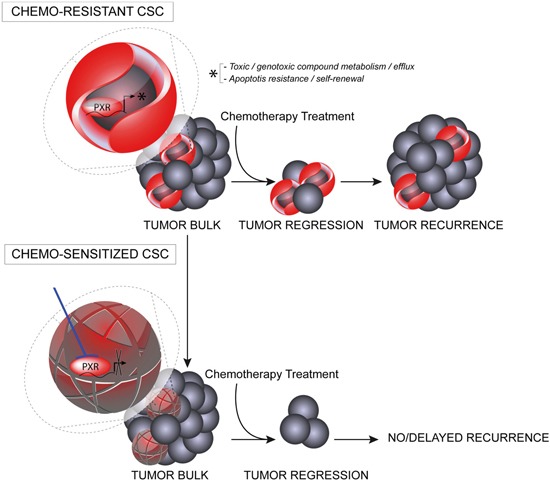
Schematic representation of putative PXR roles in CSC survival and tumor recurrence PXR expression in CSCs leads to enhanced transcription of PXR target genes promoting CSC plasticity by providing these cells with a defense arsenal that includes drug-metabolizing and transporter genes, enhanced capacity to deal with oxidative stress and genome alterations, and enhanced anti-apoptotic capabilities. Targeting PXR expression/activity using pharmacological inhibitors, may down regulate this multi-faceted protection against cytotoxic drugs and improve the efficiency of conventional chemotherapy in CRC patients through the sensitization of CSCs.

We demonstrate that PXR down-regulation decreased CSCs chemoresistance (Figure 4a) and chemotherapy-induced CSC enrichment and it also significantly delayed tumor relapse after Folfiri treatment of xenografted animals (Figure 6). In addition, the tumor-initiating ability of residual tumor cells and the proportion of CSCs found in tumor xenografts at the end of Folfiri treatment were strongly decreased in PXR knockdowns (Figure 6). These observations imply that PXR may represent a key target to improve the efficiency of conventional chemotherapy through the sensitization of CSCs and counteract the selection/emergence of chemoresistant CSCs (Figure 8). The potential implications of these results are far-reaching as post-treatment relapse is one of the major issues hampering clinical managements of CRC.

The large network of genes controlled by PXR in CSCs suggests that this receptor is a potential Achilles' heel for CSCs in colorectal cancers. Additionally, this may be true for other human cancers, as significant PXR expression levels have been detected in prostate, breast and ovarian cancers, where Aldefluor activity and ALDH1A1 expression are enriched in chemo-resistant CSCs [42–44]. Interestingly ALDH1A1 was among the most significantly deregulated genes after PXR knock-down or gain of function. Despite accumulating evidence in several cancers for the functional role of ALDH1A1 enzyme in CSC self-renewal and survival [45], the specific mechanisms involved in its regulation in CSCs remain unclear. Here we observed that PXR is an important driver of ALDH1A1 expression and activity in human colon CSC cells (Figures 5, Supplementary Figures S4 and S5). These results are in agreement with previous studies showing that Pxr activation increases both Aldh1a1 mRNA expression [20] and Pxr binding to the *Aldh1a1* gene [46] in mouse liver.

Finally, along with reports documenting the tumor-promoting [47] and chemoresistance properties [48] of PXR, and considering that Pxr knockout mice are viable, fertile and do not display major defects [49], our data point to PXR as a promising target to improve the efficiency of conventional chemotherapy. Interestingly, two molecules that were recently reported to interact synergistically with chemotherapies to eliminate CSCs, L-sulphoraphane and metformin [3, 50, 51], are non-selective PXR inhibitors [52, 53]. Thus, development of selective PXR-signaling inhibitors will represent an important step towards the validation of PXR as a clinically druggable target in comparison with these multi-targeted compounds.

## SUPPLEMENTARY MATERIALS FIGURES AND TABLES






