# Supplementary material for: Pregnane X-receptor promotes stem cell-mediated colon cancer relapse
Source: Oncotarget. 2016 Jul 18;7(35):56558–73. doi: 10.18632/oncotarget.10646 (PMC5302934; doi:10.18632/oncotarget.10646)
Supplement: Supplementary file 1 [file oncotarget-07-56558-s001.pdf]

Pregnane X-receptor promotes stem cell-mediated colon cancer relapse

SUPPLEMENTARY DATA

MATERIALS AND METHODS

Cell lines and patient-derived tumor cell culture

LS174T stable PXR transfectants (LS-PXR2 and LS-PXR6) were previously described[19]. CRC cell lines (T84, LS174T, HT29, DLD1, SW620) were obtained from ATCC and maintained in DMEM (Gibco) with 10% FBS or as spheroids in defined M11 media in ultra-low attachment flasks (Corning). M11 is DMEM/F12 (1:1) medium (Gibco), supplemented with N2, Glutamine 3 mM, Glucose 0.6%, insulin 4µg/ml (Sigma-Aldrich), Penicilin G 100U/ml, Streptomycin 100ug/ml, hBasic-FGF 10 ng/ml (R&D Systems), hEGF 20 ng/ml (R&D Systems). Patient-derived cell cultures (CRC1, 6, 14, 19, 24, 25-see S10 for clinical description) of colon cancer cells were obtained from CRC biopsies provided by CHU-Carémeau (Nîmes, France, ClinicalTrial.gov Identifier#NCT01577511) within an approved protocol. Signed informed consents were obtained from patients prior to samples acquisition in accordance with all ethical and legal aspects. Tumors were washed, minced into fragments (<2mm^3) and digested with Liberase H (0.26U/mL, Roche) and resuspended in Accumax (Sigma-Aldrich). After 2 hours at 37°C, the cell suspension was filtered through a 40µm mesh to obtain a single cell suspension and plated in DMEM medium, supplemented with FBS, glutamine, antibiotics and non-essential amino acids. When a monolayer of patient-derived tumor cells was formed, cells were detached using trypsin/EDTA and resuspended in DMEM with 10% FBS (for adherent cells) or defined M11 media (for sphere formation). Cells were cultured in a humidified atmosphere at 37°C and 5% CO_2. For PXR depleted cell lines, cells were transduced with shLUC- or shPXR-pLKO-lentiviral vectors (Sigma-Aldrich) and were selected in presence of 1µg/mL puromycin (Life Technologies).

Xenograft transplantation and chemotherapy treatment

Suspensions of 15,000 or 1,500 cells from colonospheres were injected subcutaneously into the flanks on BALB/c nu/nu (nude) in a 1:1 mixture of Matrigel and DMEM at a final volume of 100µL. Body weight and clinical signs of the mice were determined every other day. Tumor volume [(length x width x thickness)/2] was measured using calipers. Folfiri, ie. 90mg/kg Leucovorin + 50mg/kg 5-FU + 30mg/kg Irinotecan, treatment (two i.p. injection/week) was initiated once tumor volume reached 100mm^3, and mice were sacrificed when tumors reached 1500mm^3. These studies were approved by the ethics committee of the Languedoc Roussillon Region and carried out in compliance with the CNRS and INSERM ethical guidelines of animal experimentation (CEEA-LR-12051).

Patients

Liver metastasis samples, from patients with metastatic colorectal disease, were obtained according to French government regulations and with ethics committee approval (CHU Montpellier, agreement #CT 094173). Patients diagnosed with stage II or III colon cancer and treated at the Colorectal Cancer Unit of the Hospital Clínic of Barcelona between 1998 and 2005 were previously described[37]. The study was approved by the institutional Ethics Committee of the Hospital Clínic of Barcelona.

RNA extraction and gene expression analysis from paraffin embedded (FFPE) tissue blocks

Hematoxylin and eosin-stained slides and FFPE blocks of all the patients included in the study were retrieved from the Pathology Department archive of the Institut d'Investigaciones Biomèdiques August Pi i Sunyer (IDIBAPS, Barcelona, Spain). Four 10 µM thick slices were obtained from each FFPE block and used to isolate total nucleic acids by a fully automated method involving iron oxide beads coated with a nanolayer of silica on a modified VERSANT® kPCR Molecular System* (Siemens Healthcare Diagnostics, Tarrytown, NY) as previously described[37]. Samples were treated for 20 minutes with DNAse-1 and reversed-transcribed using SuperScript III reverse transcriptase (Invitrogen) using RPLO, GAPDH, PXR, ALDH1A1, 18S gene specific primers. Expression of PXR and ALDH1A1 mRNAs were analyzed by real-time qRT-PCR, blinded to patient characteristics and clinical endpoints. Expression levels were normalized to GAPDH or PRLO as endogenous control.

RNA extraction and real-time PCR

Total RNA was extracted using the RNeasy mini kit (Qiagen) and treated with DNAse-1. The first strand

cDNA was synthetized using Superscript II (Invitrogen) and random hexamers, and gene expression was measured by real-time PCR. Each cDNA sample was amplified in duplicate using SYBR Green (Roche) on the LC480 real-time PCR system. GAPDH, β-actin, RPLO, 18S ribosomal RNA and RPL13 were used as endogenous controls. All samples were normalized according to the GAPDH mRNA expression level, whose expression was the most stable and closest to our target genes. The fold changes in genes expression were calculated with the $2^{-\Delta\Delta Ct}$ method. Primer sequences are provided in Table S1.

mRNA expression profiling

RNA expression profiling of LS174T, overexpressing PXR, was performed on 44K Agilent Human Genome chip (Agilent Technologies) by competitive hybridization of each LS174T transfectant (LS-CTRL, LS-PXR2 and LS-PXR6) *versus* a pool of equal amounts of total RNA from all samples. Five microgram aliquots of total RNA from each sample and from the reference pool were used to generate labeled antisense cRNAs with T7 RNA polymerase. Reverse transcription, linear amplification, cRNA labeling, and purification were performed with the Agilent Linear Amplification kit. Hybridization was allowed to proceed for 17h at 60°C, with 1µg of cyanine 5-labeled cRNA from each sample (n=3/transfectant) mixed with the same amount of cyanine 3-labeled cRNA from the reference pool. The arrays were then washed with 0.6× and then 0.01× SSC buffers containing Triton, and were dried with a nitrogen gun before scanning with an Agilent DNA microarray scanner. The fluorescence images, thus obtained, were quantified with Feature Extraction software (Agilent Technologies).

RNA expression profiling of CRC1 ALDHbr cells transfected with PXR siRNA was performed using Affymetrix Human gene ST 2.0 DNA microarrays. $ALDH^{br}$ cell-derived CRC1 colonospheres were collected after transfection with control (siβGal) siRNA or siPXR and were analysed in biological triplicates. Preparation of cRNA was performed with the "GeneChip® WT PLUS Reagent Kit (Affymetrix)" as recommended by the supplier. Hybridizations, washes, detection and quantification were then performed as previously[57] and expression data were normalized by the Robust Multichip Average method using the Affymetrix "Expression Console" software. Analyses were performed in 'R' and its associated packages. Raw transcriptome data were deposited in ArrayExpress (http://www.ebi.ac.uk/arrayexpress/, accession number: E-MTAB-3471). Gene ontology analysis of the resulting gene list was based on GO biological processes of the Database for Annotation, Visualization and Integrated Discovery (DAVID; http://david.abcc.ncifcrf.gov/).

Gene set enrichment analysis

Gene set enrichment analysis (GSEA) was used to interrogate the similarity of genes that were differentially expressed in the microarray experiment compared to published prognosis signatures of patients. GSEA version 2-2.0.13, in pre-ranked mode, was used in these analyses. Genes were first ranked based on log fold change differential expression between PXR-overexpressing cells and control. Enrichment scores were calculated directly from probe values by constructing a custom Agilent 6x66k chip file using the chip2chip tool in the GSEA package. This chip file was used to convert gene signatures to probe signatures. We compared PXR-induced genes to gene signatures indicative of patient prognosis. Subtype B was termed the "poor prognosis" signature, conversely subtype A was termed the "good prognosis" signature. A core signature representative of each cell type was generated by taking sub sets of genes with a PAM score greater than 0.2.

Western blot

Cells were lysed and prepared for total, cytosolic or nuclear protein extractions (PIERCE) with protease inhibitors (Roche). For western blotting, samples were subjected to 10% SDS-PAGE and transferred to nitrocellulose membranes (Amersham). The following antibodies were used: GADPH (sc-32233), ALDH1A1 (sc-22589), CYP3A4 (sc-27639), and PXR (sc-48403 & sc-48340) from Santa Cruz. Bands intensities were measured on an image Image Lab software (BIORAD, version 4.1).

Luciferase and GFP reporter constructs

The pGL2b, prCYP3A4-XREMLuc (XREM CYP3A4Luc), the PXR responsive (NR1)3TKluc (PXRE TkLuc), the control pGL3TK luciferase (TKluc) plasmids and the β-galactosidase expressing plasmid, pSV-βGAL have all been described previously[24]. The EIF1αeGFP plasmid was from Addgene (#39196). The DNA fragment corresponding to the [-7600/-7200]/(-1100/+11) CYP3A4 promoter[24] was cloned in the lentiviral expression vector pLV-GFP upstream of the GFP reporter gene (from Anne Corlu, INSERM UMR991, Rennes, France).

siRNA duplexes cells transfection

Transfections were performed in 24-well plates (4 wells/condition) using Fugene-6 (Roche), Lipofectamine RNAimax and siRNA duplexes (siβGAL and siPXR) which were obtained from Invitrogen and Genecust respectively. siRNA transfections were performed according to the manufacturer's recommendation. siRNA sequences are provided in Supplementary Table 1.

In vitro cytotoxic treatment

Cells were plated at 100,000 cells/well in DMEM with 10% FBS in 6 well plates. After 48 hours cells were exposed to Firi (1X=50µM 5-FU + 500nM SN38), Folfox (50µM 5-FU + 1 µM oxaliplatin) or Firinox (50µM 5-FU + 500nM SN38 + 1 µM oxaliplatin) or vehicle (3 wells/condition). After 72 hours of treatment, cells were washed twice with ice-cold PBS before RNA extraction.

In vitro chemosensitivity assays

Cells were plated at 10^3 or 10^4 cells per well in 96-well plates in DMEM with 10% FBS. After 16 hours, cells were treated with cytotoxics for 48 to 72 hours. Cell viability was assessed by CellTiter-Glo assay (Promega). EC_{50} were calculated using Prism Software.

Aldefluor assay and fluorescence-activated cell sorting (FACS)

The Aldefluor assay (Stem Cell Technologies) was performed according to the manufacturer's instructions (Stem Cell Technologies). $ALDH^{bright}$ cells ($ALDH^{br}$) and $ALDH^{low}$ cells ($ALDH^{lo}$) were identified by comparing the same sample with and without the ALDH inhibitor diethylaminobenzaldehyde (DEAB). The gating strategy for flow cytometry analysis of Aldefluor-stained samples was as follows: cells were first stained using the Aldefluor assay, then stained with SYTOX Blue Dead Cell Stain (Invitrogen). All samples were analyzed by sequential gating including the main population (SSC vs FSC), single cells (SSC-A vs SSC-W), and viable (SYTOX Blue -negative) cells (SSC-A vs SYTOX Blue channel). Cells that had been incubated with the DEAB inhibitor were used to set the negative control gate, upon which identification of the ALDH+ subpopulation in the test samples (without DEAB inhibitor) was based. Cells were sorted using a FACSAria II (BD) and analyzed using Summit 6.0 or Cyflogic software. Dead cells were excluded based on light scatter characteristics.

Slide preparation for immunohistochemistry

After sacrifice, tumors were harvested and fixed for 2 hours with 4% PFA and were then dehydrated with serial ethanol baths followed by xylene baths before being embedded in paraffin. 6 µm slides were then cut using a microtome (Microm, HM335E). Embedded tumor slides were dewaxed by heating at 56°C and immersing in serial xylene and graded ethanol baths. Antigen retrieval was performed in boiling citrate buffer for 20 min and nonspecific binding sites were blocked for 1h in Phosphate Buffered Saline (PBS) containing 5% milk and 0,5% triton. Slides were incubated overnight at 4°C with primary antibodies diluted in blocking buffer. Anti-ALDH1A (1/100) was from BD Biosciences (60274), anti-human mitochondria (1/100) was from Millipore (MAB1273). Secondary antibodies used for immunohistochemistry were developed using 3 3′diaminobenzidine tetrahydrochloride (DAB). Slides were counterstained with hematoxylin and coverslipped with Permount. Human mitochondria- and ALDH1A1-stained areas in tumor sections were measured on an image analyzer (Image J) software.

Sphere formation assays

Percentage of Cell Forming Spheres was determined after plating 100 cells/well in M11 medium in 96 well plates in ultra-low attachment plates (Corning). Spheres were counted if their diameter exceeded 50µM. The frequency of cancer cells with *in vitro* tumorigenic potential was determined using the Extreme Limiting Dilution Analysis[29] after dilution in M11 medium of 1000/100/10 and 1 cell per well (n=18-24 wells/condition) in 96-wells. The number of wells containing at least one sphere (>50µM) was counted after 10 days.

Kaplan-Meier analyses

Disease-free survival was calculated as the time between surgical resection and confirmation of either locoregional relapse and/or distant metastasis. Development of metachronous colorectal lesions was not considered. A census of patients without tumor recurrence was taken at the last follow-up contact. Initially, expression values of evaluated genes were subjected to a received operating curve (ROC) analysis in order to choose an individual cut-off for each gene. The cut-off value was selected to minimize the distance between the curve and the upper left corner of the graph (Youden index). Thus, continuous data were converted to binary form (high and low). Kaplan-Meier curves were generated using the selected cut-off and compared according to the likelihood ratio test. p-values <0.05 were set as significant.

Statistical analysis

For each experiment, data are shown as mean ± S.E.M of at least three independent experiments. Graphpad Prism6 software was used for data analysis. The Mann Whitney test was used to analyze the difference between two groups of quantitative variables with Alpha-value set at 5%. For comparisons among three groups of quantitative variables, the Kruskal Wallis test was used. In cases where there was a significant difference between the groups, a pairwise comparison was carried out by adjusting the alpha risk using the method of Bonferroni. Student's t-tests were performed when indicated in Figure legends.

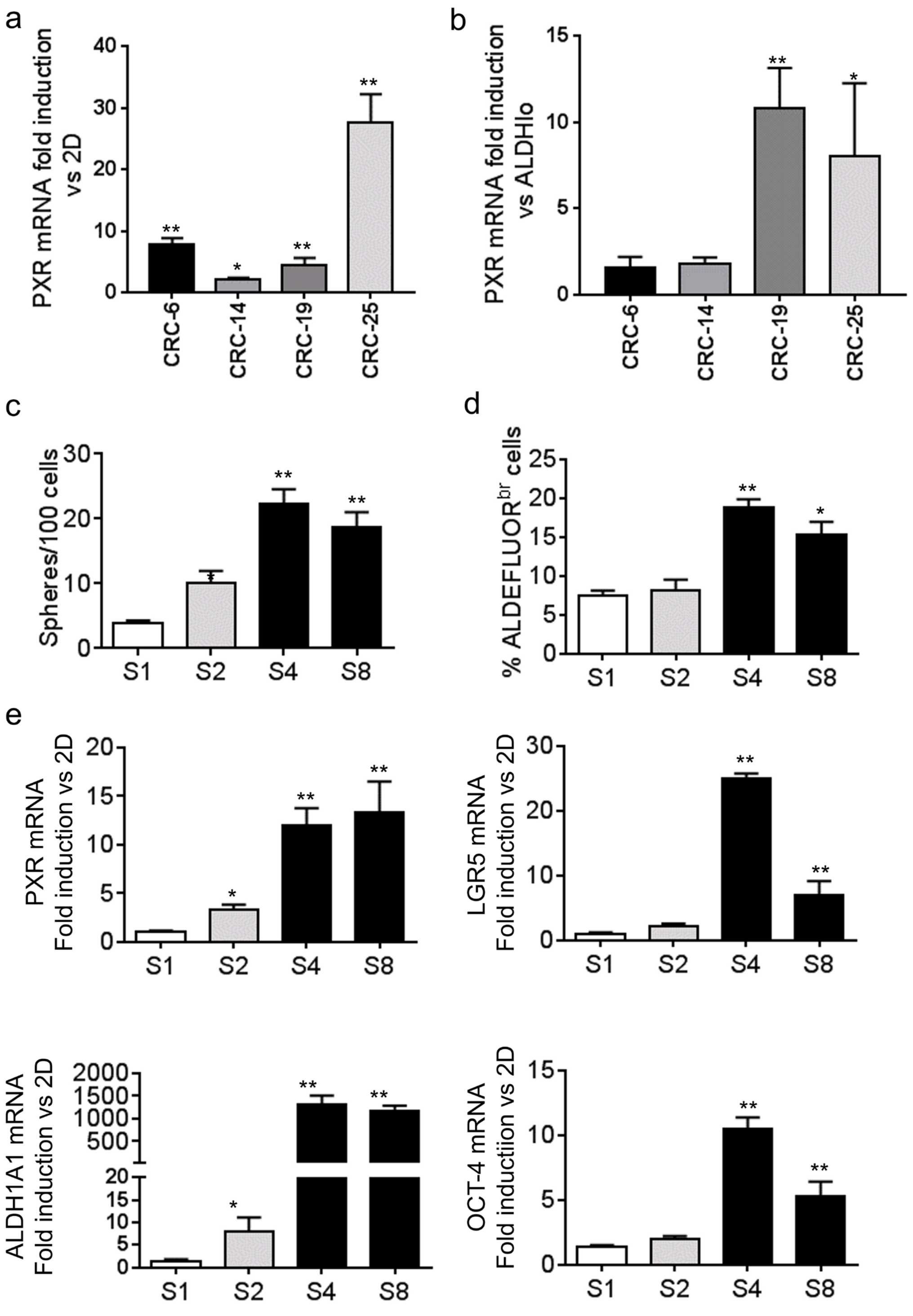

Supplementary Figure S1: PXR expression is increased in colorectal colonosphere models. a. PXR expression quantified by RT-qPCR on mRNA from four patient-derived colorectal tumor cells (CPP6, 14,19 & 25) maintained as colonospheres compared to cells grown under adherent conditions ('2D'); or **b.** after Aldefluor cell sorting (ALDHbr compared to ALDHlow). **c.** Percentage of Sphere-Forming Cells, **d.** percentage of ALDHbrcells and **e.** PXR and colon CSC marker expression quantified by RT-qPCR on mRNA from CRC1 cells maintained as colonospheres for multiple passages (S1 to S8). RT-qPCR data are expressed as mean±SEM of >3 experiments and are reported as fold change compared to cells grown under adherent conditions ('2D'). *, p<0.05; **, p<0.005 compared to 'S1'or 2D conditions.

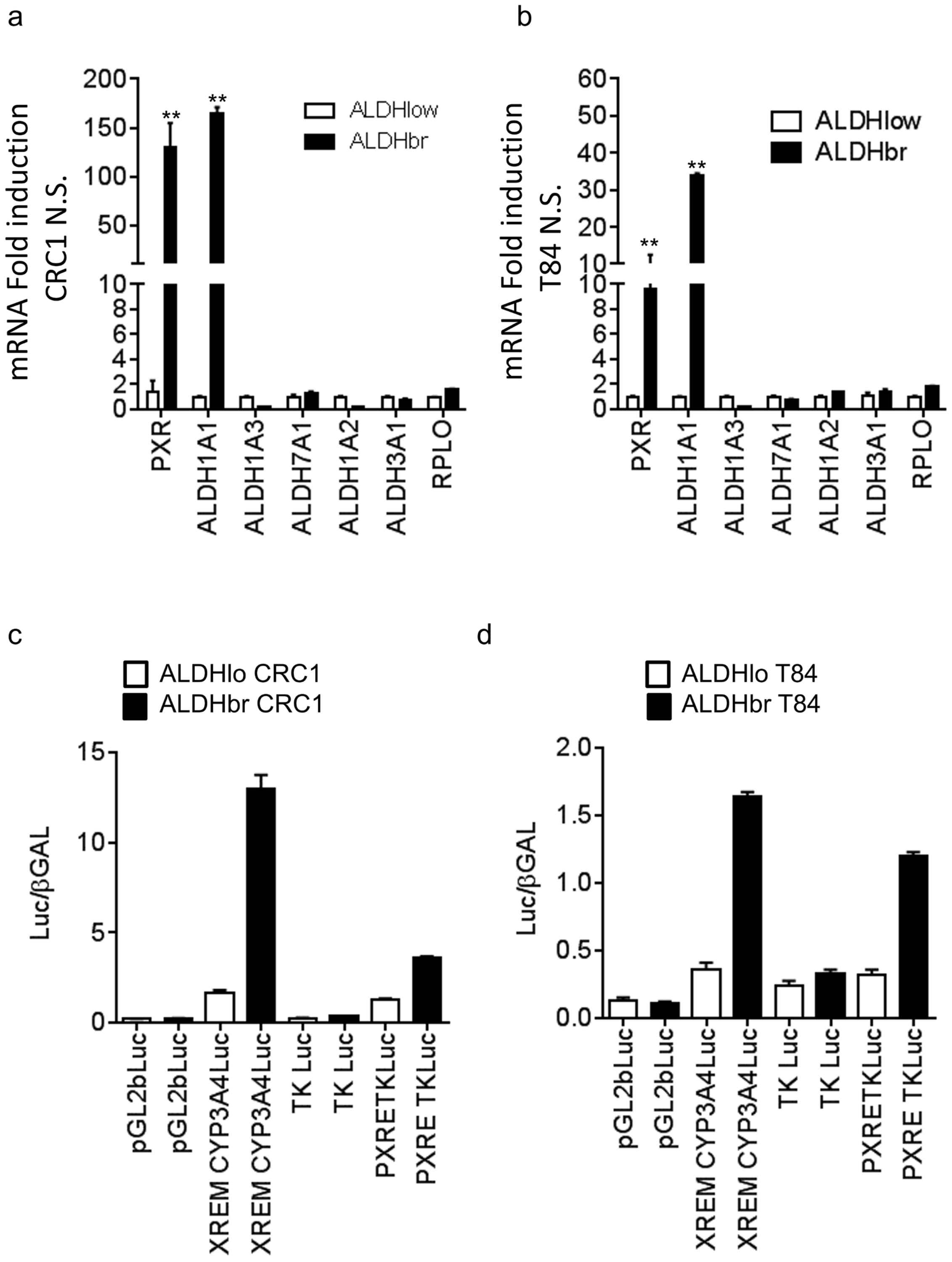

Supplementary Figure S2: PXR expression is increased in Aldefluor-positive (ALDH^{br}) cells. RT-qPCR analyses of PXR and ALDH isoforms in CRC1 **a.** or T84 **b.** ALDH^{br} and ALDH^{lo} sorted cells. Data are expressed as mean±SEM of 3 experiments. PXR transcriptional activity was measured in CRC1 **c.** and T84 **d.** ALDH^{br} and ALDH^{lo} cells. After Aldefluor-cell sorting, cells were first transfected with the indicated luciferase reporter plasmid. Luciferase and β-Galactosidase activities were measured 24 hours after transfection. Data are expressed as mean of luciferase/β-galactosidase ratio ± SEM of ≥3 experiments. *, $p<0.05$; **, $p<0.005$.

Term	up siPXR		down siPXR	
	Count	PValue	Count	PValue
GO:0009615~response to virus	14	7.42E-11		
GO:0001666~response to hypoxia	10	9.14E-06		
GO:0070482~response to oxygen levels	10	1.38E-05		
GO:0042127~regulation of cell proliferation	23	1.46E-05		
GO:0032355~response to estradiol stimulus	7	1.75E-05		
GO:0006955~immune response	21	2.24E-05		
GO:0009725~response to hormone stimulus	14	9.65E-05		
GO:0043627~response to estrogen stimulus	8	9.78E-05		
GO:0048545~response to steroid hormone stimulus	10	1.54E-04		
GO:0012501~programmed cell death	18	1.60E-04		
GO:0006952~defense response	18	1.73E-04		
GO:0009719~response to endogenous stimulus	14	2.55E-04		
GO:0008285~negative regulation of cell proliferation	13	3.23E-04		
GO:0008219~cell death	19	3.71E-04		
GO:0016265~death	19	4.03E-04		
GO:0051726~regulation of cell cycle			9	4.72E-03
GO:0008610~lipid biosynthetic process			9	4.08E-03
GO:0006333~chromatin assembly or disassembly			6	3.34E-03
GO:0022403~cell cycle phase			11	1.62E-03
GO:0051276~chromosome organization			12	1.56E-03
GO:0000279~M phase			10	1.18E-03
GO:0051301~cell division			10	5.46E-04
GO:0022402~cell cycle process			15	1.42E-04
GO:0033554~cellular response to stress			17	9.03E-06
GO:0007049~cell cycle			21	2.44E-06
GO:0006281~DNA repair			13	2.44E-06
GO:0006261~DNA-dependent DNA replication			8	3.43E-07
GO:0006974~response to DNA damage stimulus			16	2.30E-07
GO:0006260~DNA replication			16	2.28E-11
GO:0006259~DNA metabolic process			24	7.05E-12

Supplementary Figure S3: PXR drives the expression of a large number of genes that protect CSCs against genotoxic death stimuli and promote self-renewal. Significantly-enriched pathways derived through a Gene Ontology analysis of differentially-expressed genes between PXR siRNA (siPXR) and control siRNA (sibGAL)-transfected ALDH^{br} cell-derived CRC1 colonospheres.

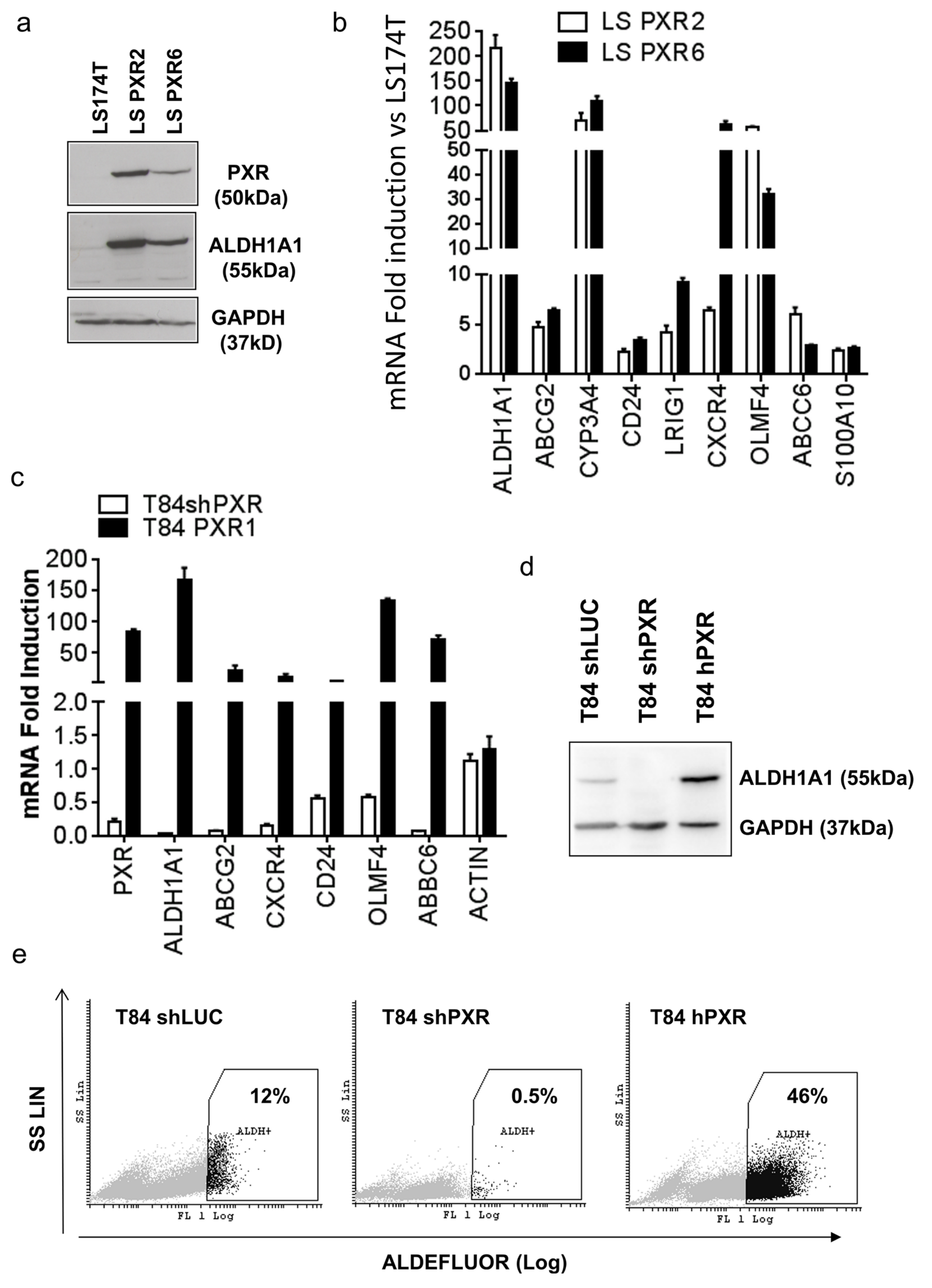

Supplementary Figure S4: PXR controls ALDH1A1 gene expression and activity in CRC cell lines. a. PXR, ALDH1A1 and GAPDH proteins were quantified by Western blotting and **b.** mRNA expression for PXR and colon CSC marker were quantified using RT-qPCR in two clones of LS174T cells over-expressing PXR (LS PXR2 and LS PXR6). Data are expressed as mean±SEM (n≥3) compared to LS174T control cells (F.I.,Fold Induction). **c.** mRNA expression for PXR and colon CSC markers was quantified using RT-qPCR in T84 cells over-expressing PXR (T84hPXR) or a PXR-targeting shRNA (T84shPXR). Data are expressed as mean±SEM of 3 experiments compared to control cells (T84 shLuc). **d.** ALDH1A1 and GAPDH proteins were quantified by Western blotting, and **e.** ALDH activity was quantified using the Aldefluor assay in T84 shLuc, T84 shPXR and T84 hPXR cells.

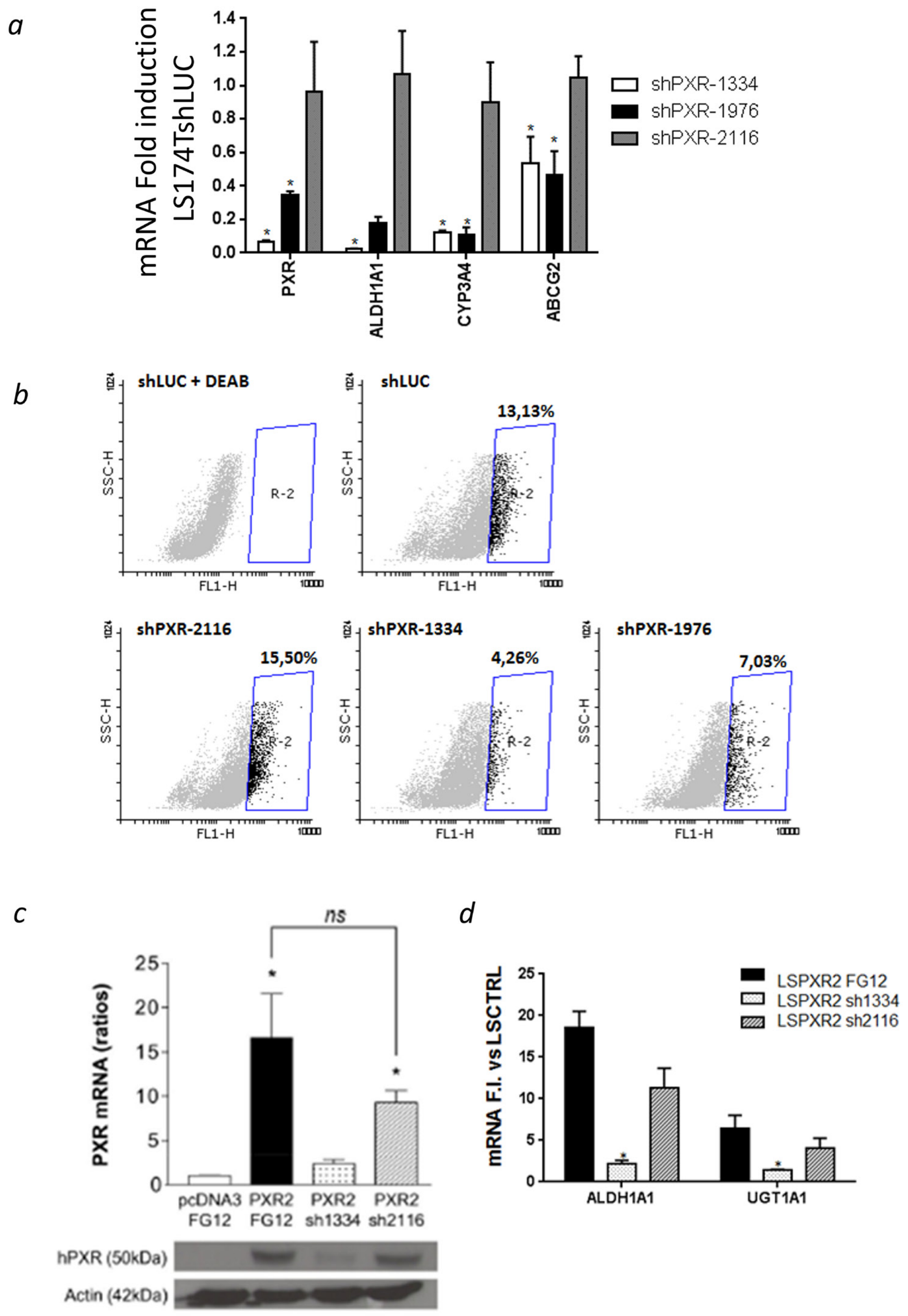

Supplementary Figure S5: Impact of effective and ineffective PXR shRNA and rescue experiment on ALDH1A1 mRNA expression and Aldefluor activity in LS174T cells. a. PXR, ALDH1A1, CYP3A4 and ABCG2 mRNA expression in LS174T cells transduced with effective (sh1334 & sh1976) or ineffective (sh2116) PXR-targeting shRNAs or shLUC control. Data are expressed as mean±SEM of 4 experiments compared to LS174shLUC (F.I., Fold Induction). *p<0.05 (student's t-test) compared to control LS174TshLUC. **b.** Aldefluor analysis of LS174T cells transduced with effective (sh1334 & sh1976) or ineffective (sh2116) PXR-targeting shRNAs or shLUC control. Percentage of ALDHbr cells are indicated in each dot plot profile. **c.** PXR mRNA and protein expression, and **d.** expression of ALDH1A1 and UGT1A1 mRNAs in control LS174T cells (expressing pcDNA3.1) or in LS174T cells overexpressing human PXR (LSPXR2) transduced with effective (sh1334) or ineffective (sh2116) PXR-targeting shRNAs or control FG12 vector. Data are expressed as mean±SEM of 3 experiments compared to LS174 control cells (F.I.,Fold Induction). *p<0.05 (student's t-test) compared to LSPXR2 transduced with FG12 vector.

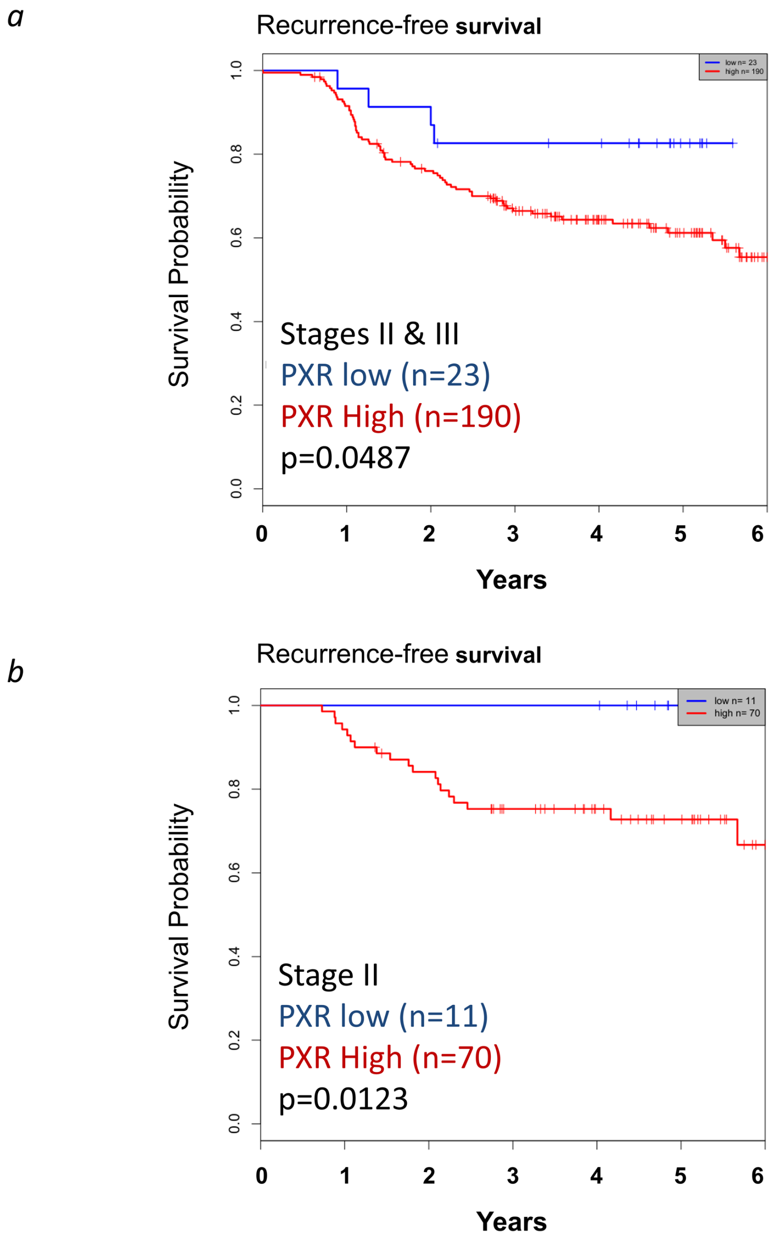

Supplementary Figure S6: PXR is a poor prognosis factor in colon cancer patients treated with 5-FU-based chemotherapy. Kaplan-Meier estimates of probability of being free of tumor recurrence according to PXR expression level normalized according to GAPDH mRNA expression in CCR primary tumors of stage II & III **a.**, or stage II **b.** patients treated with 5-FU-based chemotherapy. Patients with low gene expression are depicted in blue and patients with high gene expression are depicted in red, P-value from the likelihood ratio test is shown in each case.

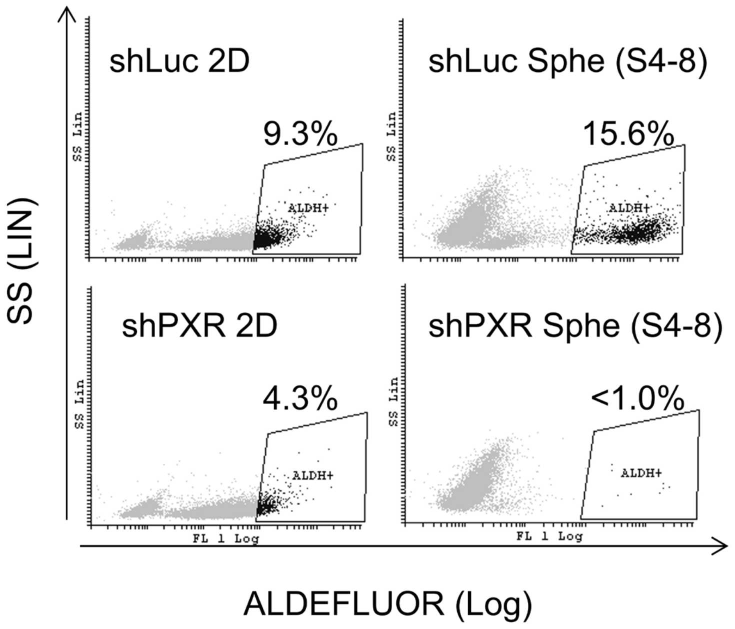

Supplementary Figure S7: Knock-down of PXR alters the self-renewal of colorectal CSCs *in vitro.* Percentage of ALDH^{br} cells using the Aldefluor assay in shLuc and shPXR transduced CRC1 colon cancer cells maintained in adherent conditions ('2D') or as colonospheres during 4 to 8 passages ('Sphe').

a

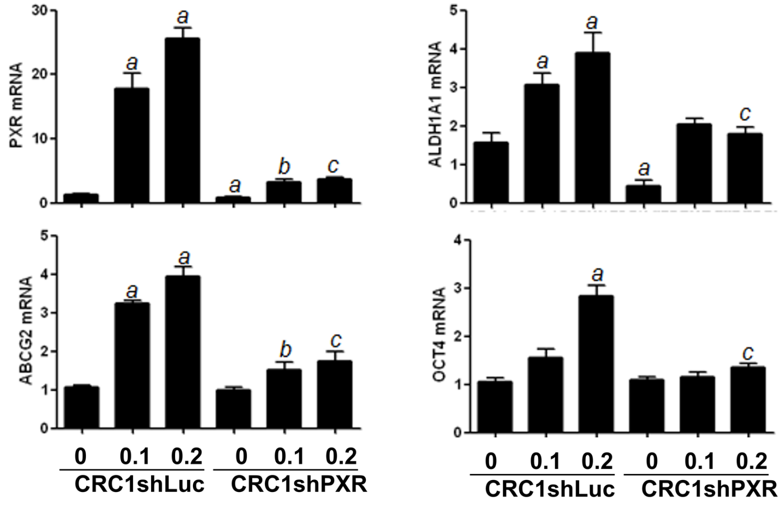

b

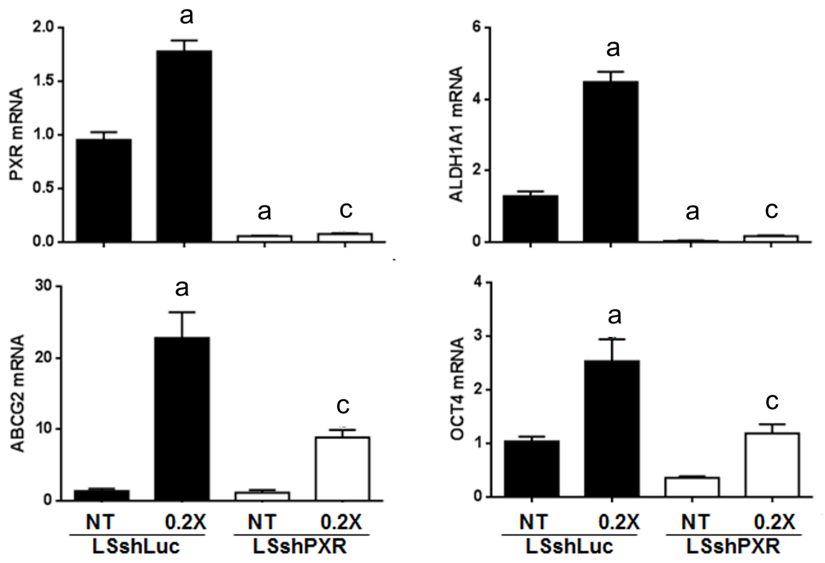

Supplementary Figure S8: PXR depletion in CRC cell lines impairs chemotherapy-induced enrichment of PXR and CSC markers *in vitro*. Relative mRNA expression of PXR and colon CSC markers in CRC1 **a.** or LS174T **b.** cells expressing control (shLuc) or PXR-targeting (shPXR) shRNA exposed for 72 hours to vehicle (NT) or the indicated dilutions of Firi (1X=50µM 5-FU + 500nM SN38). Data are expressed as mean±SEM of ≥4 experiments and compared to shLuc cells, “a”, p<0.05 compared to untreated shLuc cells; “b”, p<0.05 compared to 0.1X Firi-treated shLuc cells, “c”, p<0.05 compared to 0.2X Firi-treated shLuc cells.

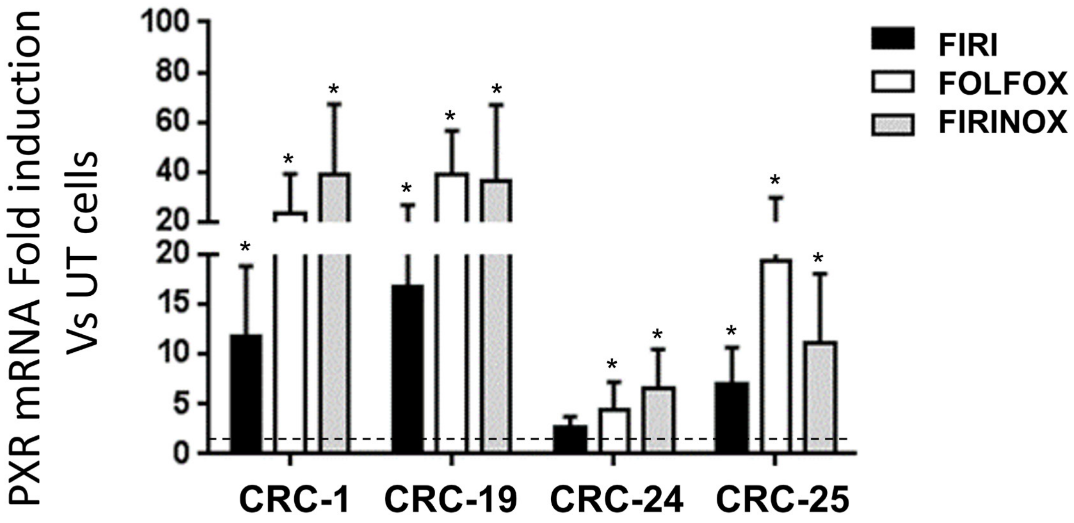

Supplementary Figure S9: PXR expression enrichment following chemotherapies in four patient-derived colon cancer cells. PXR mRNA expression in established colorectal cancer cell lines from 4 different patients maintained in adherent conditions and exposed for 72 hours to vehicle (UT) or to Firi (50 µM 5-FU + 500nM SN38), Folfox (50µM 5-FU + 1 µM oxaliplatin) or Firinox (50µM 5-FU + 500nM SN38 + 1 µM oxaliplatin) treatments. Data are expressed as mean±SD of 2 experiments performed in triplicates and are normalized according to UT cells; *$p<0.05$.

Clinical characteristics of patient

	CRC-1	CRC-6	CRC-14	CRC-19	CRC-25
Gender	M	M	F	M	M
Age	76	71	65	65	77
Location of tumor	Transverse colon	sigmoid	descending colon	hepatic metastasis	rectum
KRAS status	WT	WT	Mut	Mut	WT
TNM classification	T4N0Mx	T3N2aM1	T2N0M0	TXN+M1	T3bN1M0
Radiotherapy	No	No	No	No	Yes
Chemotherapy	unknown	Bevacizumab folfiri	none	Bevacizumab folfiri	Bevacizumab Cetuximab folfiri

Supplementary Figure S10: Clinical information of CRC patient derived cell lines. Clinical information indicates tumor location, KRAS mutational status, TNM classification and the use of radio- and/or chemotherapy.

Supplementary Table S1: primer and siPXR sequences

Primers list

Genes	Primers	Sequences (5'-3')
**β-Actin**	forward	AGCACGGCATCGTCACCAACT
	reverse	TGGCTGGGGTGTTGAAGGTCT
**RPL13**	forward	CATAGGAAGCTGGGAGCAAG
	reverse	GCCCTCCAATCAGTCTTCTG
**18S**	forward	GTAACCCGTTGAACCCCATT
	reverse	CCATCCAATCGGTAGTAGCG
**GAPDH**	forward	AATTGAGCCCGCAGCCTCCC
	reverse	CCAGGCGCCCAATACGACCA
**RPLO**	forward	TCGACAATGGCAGCATCTAC
	reverse	GCCTTGACCTTTTCAGCAAG
**UGT1A1**	forward	AAATCCACTATCCCAGGAAT
	reverse	AGTATCGTGTTGTTCGCAAG
**ABCG2**	forward	GGCTTTCTACCTGCACGAAAACCAGTTGAG
	reverse	ATGGCGTTGAGACCAG
**PXR**	forward	TCCGGAAAGATCTGTGCTCT
	reverse	AGGGAGATCTGGTCCTCGAT
**CXCR4**	forward	TGACGGACAAGTACAGGCTGC
	reverse	CCAGAAGGGAAGCGTGATGA
**LGR5**	forward	CTTCCAACCTCAGCGTCTTC
	reverse	TTTCCCGCAAGACGTAACTC
**oct-04**	forward	GTGGAGAGCAACTCCGATG
	reverse	TGCTCCAGCTTCTCCTTCTC
**OLFM4**	*forward*	AAGCTGCAGGGGATTTGGGGG
	reverse	AGGTCCCACGGTCATCCACG
**ABCC6**	*forward*	GCTGGGACCCACGACGACAG
	reverse	GGGGTACCCAGACCCCTGCT
**TERT**	*forward*	CAGGGCTCCATCCTCTCCAC
	reverse	TGATGGAGGTCCGGGCATA
**TNFRSF11A**	*forward*	CGGCTGGGTACCACTGGAGC
	reverse	TGCAAGGCAAGGTTTGCACACTG
**ALDH1A1**	forward	TCCTGGTTATGGGCCTACAG
	reverse	CAACAGCATTGTCCAAGTCG
**CYP3A4**	forward	TATTCTGTCTTCACAAACCG
	reverse	TTTCTCACCAACACATCTCC
**siRNA sequences**		
**siPXR**	*1976*	GAUUCAACACAGUGUUCAATT
	1334	GGAGUUGUUCGGCAUCACATT
	2116	GGAGGGCCAUGAAACGCAAAT

Supplementary Table S2: deregulated gene list in CRC1 ALDHbr transfected with sibGAL or siPXR. F.C.= Fold change compared to control sibGAL transfected cells

See Supplementary File 1

Supplementary Table S3: deregulated gene list in LS174T cell populations overexpressing human PXR (LSPXR2 and LSPXR6). F.C.= Fold change compared to control LS174T cells

See Supplementary File 2
